# Prevalence of Dyslipidemia in Tibetan Monks from Gansu Province, Northwest China

**DOI:** 10.1515/biol-2020-0016

**Published:** 2020-05-07

**Authors:** Yan Fang, Xing-hui Li, Yan Qiao, Nan Wang, Ping Xie, Gang Zhou, Peng Su, Hui-yuan Ma, Ji-yang Song

**Affiliations:** 1 Department of Cardiology, Gansu Provincial Hospital, No. 204 Donggang West Road, Lanzhou, Gansu, 730000, China

**Keywords:** atherosclerotic cardiovascular diseases, dyslipidemia, low-density lipoprotein cholesterol, high-density lipoprotein cholesterol, triglycerides, body mass index, Tibetan monks

## Abstract

Tibetan monks have a special way of life and food habits, however, little is known about their dyslipidemia. This study aimed to investigate the prevalence of dyslipidemia and risk factors of this population. A cross-sectional study of dyslipidemia was conducted in 876 Tibetan monks and 912 local residents in the same area. All subjects underwent interviews and physical examinations. The total cholesterol (TC), low-density lipoprotein cholesterol (LDL-C), high-density lipoprotein cholesterol (HDL-C), and triglycerides (TG) of the subjects were analyzed. Compared to local residents, the overall prevalence of dyslipidemia in monks was 29.5%, which was significantly lower (p<0.05). It was increased with higher age and BMI, but reduced with higher educational level (p<0.05). The typical forms of dyslipidemia in monks were elevated TG and low HDL-C, while it was lower HDL-C in residents (p<0.05). Our study demonstrated that monks in Gannan Tibetan autonomous district had a lower prevalence of dyslipidemia. It suggested that the relatively healthy lifestyle and food habits of monks were mainly responsible of the lower prevalence of dyslipidemia.

## 1 Introduction

The morbidity and mortality of atherosclerotic cardiovascular diseases (ASCVD) have increased dramatically with the development of economy and progression of urbanization and industrialization [1]. Dyslipidemia plays a major role in the expansion and progression of atherosclerosis and has been recognized as one of the most important modifiable risk factors for cardiovascular disease. The typical forms of dyslipidemia include elevated total cholesterol (TC), elevated low-density lipoprotein cholesterol (LDL-C), reduced high-density lipoprotein cholesterol (HDL-C) and elevated triglycerides (TG). The risk of mixed forms of dyslipidemia is growing due to the changes in lifestyle, including diet patterns and degree of physical activity [2]. The relationship between dyslipidemia and other non-communicable diseases such as hypertension, diabetes mellitus, obesity, and overweight has been evaluated in previous studies [3, 4, 5, 6]. In the recent years, an increasing trend in the prevalence of dyslipidemia has been observed in adults among different populations in China [7]. However, the prevalence of dyslipidemia in Tibetan monks is unclear. Tibetan monks become so in childhood. They are not allowed to get married and will spend almost their entire lifetimes in the temple. They have a special way of life and food habits, such as exercising and chanting frequently, mostly abstaining from eating meat, and abstaining from alcohol use. The aim of this study was to investigate the prevalence of dyslipidemia and risk factors of Tibetan monks living in Gansu Province, Northwest China.

## 2 Experimental Procedures

### 2.1 Demographic information

During May to July 2016, a total of 980 monks aged 18 to 70 years were randomly selected from 5 temples in the Gannan Tibetan autonomous district of Gansu Province, of whom 876 completed the survey. A total of 1034 Tibetan residents living in a local district between the age of 18 and 70 years were simultaneously surveyed and 912 of them completed the survey. The sample size was calculated based on the formula n =ｕ^2^π(1-π)/δ^2^ to calculate. π is 0.30, which refers to the prevalence rate of the epidemiology survey of 2016. u is 1.96, which refers to the z-score for confidence level 95% of normal distribution. δ is the tolerances, which equals to the prevalence rate multiply with the sampling error (0.15). The minimum sample size is 405 (=[1.962×0.30(1-0.30]/(0.30×0.15)^2^). We excluded monks younger than 18 and older than 70; those with abnormal renal and liver function; those with mental illness; and those with confirmed cancer.

Concentrated investigation and household visits were performed. All participants completed a questionnaire-based interview including age, residential area, education, smoking, alcohol consumption, dietary assessment, physical activity, and history of dyslipidemia in the past one year. The anthropometric check was carried out to measure height and weight. Body mass index (BMI) was subsequently calculated as weight in kilograms divided by the square of height in meters. BMIs were categorized into 4 groups which were underweight (<18.5 kg/m^2^), normal weight (18.5-23.9 kg/m^2^), overweight (24-27.9 kg/m^2^), and obesity (≥28 kg/m^2^), based on the Chinese criteria [9]. The age groups were classified into young (18-39 years), middle-aged (40-59 years) and older-aged (≥60 years). Educational level groups were categorized into unschooled, primary and middle school, and above. Smoking was defined as currently smoking or having at least one cigarette per day for at least 6 months. Alcohol consumption was defined as drinking at least once per week over the last 12 months. The 24-h dietary recall method was used to record food habits which included food names, quantity and cooking method, and the intakes of macronutrients from the ingredients calculated by using the 2002 Chinese Food Composition Table [10]. Physical activity was assessed with the use of questions designed to measure work activities taking at least 30 minutes daily for 6 months or above. The heavier activities were defined as transporting and loading heavier goods, digging, shoveling, running, and playing basketball and football, etc. The moderate activities were defined as driving a vehicle, installing electrical appliance, cutting metal, sword dancing, and Taiji boxing. The lighter activities were defined as cooking, washing, sweeping, mopping, wiping, and walking.

**Informed consent**: Informed consent has been obtained from all individuals included in this study.

**Ethical approval**: The research related to human use has been complied with all the relevant national regulations, institutional policies and in accordance the tenets of the Helsinki Declaration, and has been approved by the Institutional Ethical Committee of Gansu Provincial Hospital.

### 2.2 Diagnostic Criteria

Blood samples were collected from an antecubital vein into vacutainer tubes containing ethylene diamine tetraacetic acid (EDTA) in the morning after at least 12 h of fasting. TC, LDL-C, HDL-C and TG were analyzed by an automatic biochemical analyzer (Abbott Architect C16000, Abbott Laboratories, USA) under instruction. All the samples were blindly duplicated. According to the Chinese guidelines on the prevention and treatment of dyslipidemia in adults (2007) [8], dyslipidemia was defined as the appearance of high TC, and/or high LDL-C, and/or low HDL-C and/or high TG, and/or having a history of dyslipidemia disease in the past one year, and/or currently receiving treatment with lipid-lowering medications. High levels of TC, LDL-C and TG were determined when the value was more than 6.22 mmol/L (240 mg/dL), 4.14 mmol/L (160 mg/dL), and 2.26 mmol/L (200 mg/dL), respectively. Low level of HDL-C was confirmed when the value was less than 1.04 mmol/L (40 mg/dL).

### 2.3 Statistical Analysis

Continuous variables were reported as mean values plus standard deviations, and categorical variables were reported as numbers and percentages. Differences between different groups were evaluated using Student’s t-test, ANOVA, and the χ^2^-test by performing SPSS version 19.0 software. P values less than 0.05 were considered as statistically significant.

## 3 Results

### 3.1 Demographic and Lifestyle Characteristics

During May to July 2016, 876 Tibetan monks and 912 residents completed surveys for this study. The demographic and lifestyle characteristics of the subjects are shown in Table 1. The monks were all males because of local religious custom; all control participants selected were also males. The average age of Tibetan monks and residents was 41.30±9.32 years and 42.14±8.67 years, respectively. No statistical significance was found in the average age, age groups, and heavier activity between the two populations (p>0.05). However, compared to residents (23.97±0.22 kg/m^2^), the average BMI of monks was lower: 22.01±0.18 kg/m^2^ (p=0.04). More monks had normal weight (75.9% vs 65.9%, p<0.01) and moderate activity (57.1% vs 47.6%, p<0.01). Furthermore, less monks were overweight (12.9% vs 19.2%, p<0.01), obese (8.2% vs 13.8%, p<0.01), and had lighter activity (22.9% vs 33.5%, p<0.01) and consumed cigarettes and alcohol (p<0.01). The overall education level of monks was lower than that of residents: 481 (54.9%) monks were unschooled, while 457 (50.1%) and 151 (16.6%) of residents attended primary school and middle school and above, respectively.

**Table 1:** Demographic Characteristics and Lifestyle Factors of Adults in Tibetan Residents and Monks in Gansu Province, Northwest China

Characteristics	Tibetan residents	Tibetan monks (n=912)	p value (n=876)
Age mean (years)	42.14±8.67	41.30±9.32	0.160
Age group (years)			
18-39(n(%))	486(53.3)	451(51.5)	0.554
40-59(n(%))	273(29.9)	254(29.0)	0.402
≥60(n(%))	153(16.8)	171(19.5)	0.116
BMI mean (kg/m2)	23.97±0.22	22.01±0.18	0.045
＜18.5(n(%))	10(1.1)	26(3.0)	0.092
18.5-24(n(%))	601(65.9)	665(75.9)	0.006
24-28(n(%))	175(19.2)	113(12.9)	0.008
＞28(n(%))	126(13.8)	72(8.2)	0.009
Educational level			
Unschooled(n(%))	304(33.3)	481(54.9)	0.004
Primary(n(%))	457(50.1)	302(34.5)	0.005
Middle school and above(n(%))	151(16.6)	93(10.6)	0.009
Cigarette smoking(n(%))	335(36.7)	8(0.9)	0.000
Alcohol consumption(n(%))	552(63.0)	28(3.2)	0.000
Physical activity(h/day)			
Heavier(n(%))	172(18.9)	175(20.0)	0.157
Moderate(n(%))	434(47.6)	500(57.1)	0.008
Lighter(n(%))	306(33.5)	201(22.9)	0.009

BMI : body mass index.

### 3.2 Dietary Characteristic

Both Tibetan monks and residents consumed a mixed diet, but the intake of ingredients varied significantly (Table 2). When compared to local residents, monks consumed significantly less fruit, meat, eggs, milk, butter, cheese, oil and sugar, but more flour, rice and vegetables (p<0.05). In contrast, the intake of total energy, fat and protein were higher in the resident group than in the monk group (p<0.01).

**Table 2:** Average Diet of Adults in Tibetan Residents and Monks in Gansu Province, Northwest China

Diet	Tibetan residents	Tibetan monks	p value
Flour (g/day)	36.0±7.6	45.8±8.8	0.009
Rice (g/day)	20.0±5.0	26.4±4.6	0.036
Vegetables (g/day)	202.5±10.0	257.2±12.8	0.008
Fruit (g/day)	81.8±4.5	60.3±5.6	0.004
Meat (g/day)	134.7±11.8	26.9±5.0	0.000
Egg (g/day)	23.9±2.4	8.8±2.0	0.001
Milk (ml/day)	325.2±25.0	230.6±12.2	0.020
Butter (g/day)	95.8±9.0	38.5±7.8	0.003
Cheese (g/day)	67.2±5.9	20.5±6.0	0.000
Oil(ml/day)	16.8±2.0	10.0±1.4	0.032
Sugar (g/day)	87.6±5.1	64.8±3.9	0.005
Energy(kJ/day)	9432.5±283.4	7557.8±220.6	0.001
Carbohydrate(% of energy)	39.0±3.4	66.1 ±3.0	0.002
Fat(% of energy)	38.7±3.0	21.0±2.7	0.008
Animal Fat(% of energy)	23.1±3.2	7.0±1.8	0.000
Protein(% of energy)	22.3±2.2	12.9±2.0	0.037
Animal protein(% of energy)	13.7±1.8	7.8±1.9	0.006

### 3.3 Variation of Lipid Levels between Monks and Residents

Table 3 showed the mean lipid levels of all participants within specific age, BMI and educational level groups. The monks had lower average TC and LDL-C levels than Tibetan residents in young (18-39 years old) and middle-aged (40-59 years old) groups, BMI of 18.5-23.9 kg/m^2^ and 24-27.9 kg/m^2^ groups, and in lower educational level groups (p<0.05). Higher average TG levels were found in monks from middle- and older-aged group, the overweight and obesity group, and also in the lower educational level group (p<0.05). No significant difference of HDL-C level was showed between monks and residents (p>0.05).

**Table 3:** Deviation of Lipid Profiles by Age, BMI and Education Level Group for Adults in Tibetan Residents and Monks in Gansu Province, Northwest China

		Tibetan residents (mmol/L)				Tibetan monks (mmol/L)			p value
	TC	LDL-C	HDL-C	TG	TC	LDL-C	HDL-C	TG	
**Age group (years)**									
18-39	4.98±0.83	3.25±0.96	1.20±0.36	1.68±0.40	4.70±0.79	3.12±0.86	1.12±0.28	1.64±0.40	<0.05
40-59	5.23±0.74	3.40±0.82	1.11±0.28	1.70±0.44	5.12±0.65	3.21±0.80	1.08±0.30	1.74±0.38	<0.01
≥60	5.20±1.02	3.38±0.92	1.10±0.30	1.67±0.38	5.14±0.86	3.30±0.72	1.10±0.27	1.71±0.41	<0.05
BMI group **(kg/m^2^)**									
＜18.5	4.06±0.53	2.85±0.75	1.00±0.34	1.58±0.42	3.96±0.45	2.72±0.66	1.10±0.26	1.44±0.36	<0.05
18.5-24	5.32±0.67	3.33±0.74	1.05±0.26	1.66±0.37	5.19±0.58	3.24±0.76	1.06±0.30	1.65±0.34	<0.05
24-28	5.43±0.82	3.45±0.85	1.02±0.26	1.84±0.48	5.37±0.66	3.41±0.82	0.99±0.27	1.70±0.30	<0.05
＞28	5.70±1.14	3.50±0.90	0.96±0.28	2.05±0.52	5.68±0.78	3.43±0.78	0.95±0.29	1.89±0.46	<0.05
Educational level group									
Unschooled	5.40±0.80	3.35±0.76	1.14±0.38	1.78±0.42	5.28±0.76	3.22±0.66	1.12±0.26	1.70±0.42	<0.01
Primary	5.34±0.71	3.42±0.72	1.16±0.30	1.65±0.34	5.22±0.66	3.31±0.70	1.18±0.27	1.64±0.36	<0.05
Middle school and above									
	5.30±0.72	3.28±0.80	1.20±0.32	1.63±0.35	5.34±0.76	3.13±0.62	1.10±0.20	1.60±0.33	0.20

TC: total cholesterol, LDL-C: low-density lipoprotein cholesterol, HDL: high-density lipoprotein cholesterol, TG: triglycerides.

### 3.4 Prevalence of Dyslipidemia

**Figure 1:**
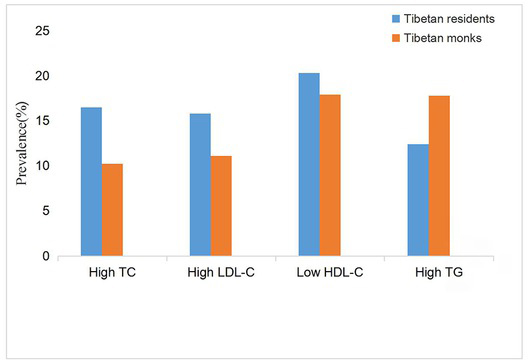
The overall prevalence of high TC, high LDL-C, low HDL-C and high TG in monks and residents.

The overall prevalence of high TC, high LDL-C, low HDL-C and high TG in monks and residents were 10.2% vs 16.5% (P<0.01), 11.1% vs 15.8% (P<0.01), 17.9% vs 20.3% (P>0.05), and 17.8% vs 12.4% (P<0.05), respectively (Figure 1). The prevalence of dyslipidemia in the two populations with specific age, BMI and educational levels was analyzed (Table 4). Monks had lower overall prevalence of dyslipidemia than local residents (29.5% vs 37.9%, p<0.05). For both populations, the prevalence of dyslipidemia increased with age, higher BMI, and lower education level.

**Table 4:** Prevalence of Dyslipidemia by Age, BMI and Education Level Group for Adults in Tibetan Residents and Monks in Gansu Province, Northwest China

	Tibetan residents Prevalence(n(%))	Tibetan monks Prevalence(n(%))	p value
Age group (years)			
18-39	152(31.3)	105(23.3)	0.007
40-59	110(40.1)	83(32.7)	0.009
≥60	84(54.9)	70(40.9)	0.005
BMI group (kg/m2)			
＜18.5	2(20.0)	5(19.2)	0.431
18.5-24	120(20.0)	125(18.8)	0.382
24-28	114(65.1)	68(60.2)	0.043
＞28	110(87.3)	60(83.3)	0.039
Educational level group			
Unschooled	138(45.4)	147(30.6)	0.004
Primary	160(35.0)	84(27.9)	0.006
Middle school and above	48(31.8)	27(29.0)	0.220
Total	346(37.9)	258(29.5)	0.023

BMI: body mass index.

## 4 Discussion and Conclusions

Dyslipidemia is one of the significant risk factors for coronary heart disease besides age, family history, cigarette smoking, diabetes mellitus, and hypertension [11]. The prevalence of dyslipidemia varies widely according to the ethnic, socioeconomic and cultural characteristics of distinct population groups [12]. The level of serum lipids in the Chinese population has greatly increased since improved economic development was associated with changes in ways of life and food habits [13]. A survey in China showed that the geographical distribution characteristics of dyslipidemia was higher in north than in south, and higher in the coastal area than in the inland region [14]. The Chinese national nutrition and health survey (CNHS) reported that the national average prevalence of dyslipidemia was 18.6% in 2002 [15]. Wu et al. reported that 36.5% of the population in Shanghai, one of the places with the most developed economy in China, had dyslipidemia in 2003 [4]. In Beijing, the capital city of China, there were 35.4% adults had dyslipidemia in 2008 [3]. Jun et al. found that the prevalence of dyslipidemia in Han, Uygur and Kazak populations in the Xinjiang Uygur Autonomous region were 58.58%, 48.27% and 49.60%, respectively [16]. We showed for the first time the prevalence of dyslipidemia in Tibetan residents was 37.9%, which was similar to the aforementioned studies. However, the prevalence of Tibetan monks was 29.5%, which was significantly lower than Tibetan residents.

As one of the minority nationalities, the Tibetan residents have their own genetic characteristics, lifestyle, and religious beliefs. They live in high altitude regions and traditionally consume a diet with more meat, butter, sugar and oil, which contained a higher proportion of saturated fatty acids and salt [17, 18]. Because of the colder weather, the residents have less exercise and drink strong wine. However, the monks live in monasteries and experienced a relatively simper life since they were young. According to their religious rules, they are unmarried, abstain from drinking and smoking, are strict vegetarians, and consume less sodium and lower saturated fatty acids in the diet. Furthermore, the monks have more moderate physical exercise than local residents [19, 20]. It is possible that the different customs between monks and residents mainly contributed to the difference in prevalence of dyslipidemia in the two populations within the same area.

It has been previously reported that the prevalence of dyslipidemia increased with age [21, 22, 23, 24, 25, 26], which were consistent with our study. We found that mean lipid concentration levels increased from 18-to-59-year-old group, but did not increase further in older participants. The effects of age on lipid levels are not exactly known. With the aging population trend in China, more individuals may be affected by dyslipidemia. In addition, our findings showed that the mean levels of lipids and prevalence of dyslipidemia increased with BMI, which also was consistent with previous studies [23, 27, 28]. Compared with the local residents, the percentage of the overweight and obese was relatively lower in monks. The prevalence of dyslipidemia was significantly lower in subjects who achieved higher education than those illiterate or less schooled. It is possible that people having higher education level were more aware of their health, and tended to seek early management of any adverse health condition [29, 30]. This trend was demonstrated in Tibetans residents, but not obvious among monks. We speculate that the stronger religious belief had an important influence than other factors on dyslipidemia of Tibetan monks.

In this study, 10.2% monks had elevated TC, 11.1% had elevated LDL-C, 17.9% had decreased HDL-C, and 17.8% had elevated TG concentrations. These parameters were 16.5%, 15.8%, 20.3% and 12.4%, respectively, for local residents. The prevalence of high TG was greater in monks, while the prevalence of low HDL-C was greater in residents. It has been reported that low HDL-cholesterol combined with high TG could dramatically increase the risk of CHD [31, 32, 33]. The lipid levels in different regions may be influenced by economic development, urbanization, dietary pattern changes in transitional periods, and possibly genetic susceptibility [34, 35]. The subjects who lived in remote and poverty areas may not undergo clinical detection and treatment since dyslipidemia is almost asymptomatic. Furthermore, some patients who rejected to see a doctor believed that the disease was predestined by Buddha. Therefore, the monks still faced the challenges of dyslipidemia although they had a relatively lower prevalence of this disease.

There were some limitations of our study. Firstly, all monks are male, unmarried, abstained from smoking and drinking, because of religious rules. Therefore, we did not analyze the prevalence of dyslipidemia by reported risk factors such as gender, smoking and drinking. Secondly, the sample capacity was relatively small because of religious regulations and resource constraints. Finally, other risk factors (such as stress, genetic factors) were not analyzed in the study.

In conclusion, our study showed that the prevalence of dyslipidemia was lower in Tibetan monks than in local residents, which could be related to their ways of diet and lifestyle. Moreover, it suggested that the result of this disease would become more serious in consideration of the religious influence and relatively lower level of economic development. Comprehensive strategies, such as training the local Tibetan doctor and increasing public awareness of dyslipidemia, are needed in the future to prevent the harm of this condition.
